# An Epigenetic Prism to Norms and Values

**DOI:** 10.3389/fgene.2018.00063

**Published:** 2018-02-26

**Authors:** Kim Hendrickx, Ine Van Hoyweghen

**Affiliations:** ^1^Fonds Wetenschappelijk Onderzoek, Brussels, Belgium; ^2^Life Sciences and Society Lab, Centre for Sociological Research (CeSO), KU Leuven, Leuven, Belgium

**Keywords:** epigenetics, (post-)ELSI, norms, values, relationality, ecology

## Abstract

In this article, we ask to what extent the specific characteristics of epigenetics may affect the type of questions one can ask about human society. We pay particular attention to the way epigenetic research stirs debate about normative and moral issues. Are these issues implied by scientific evidence as an outcome of research? Or do moral and normative issues also shape how research is done and which problems it addresses? We briefly explore these questions through examples and discussions in (social-) scientific literature. In the final section, we propose an additional dimension and a refocusing of attention from issues of scientific evidence alone (asking what kind of evidence epigenetics produces and how it does so) to a broader picture on epigenetics as a mode of attention that encourages relational and process-oriented thinking with entities, values and scales that may not yet fit within conventional problem-frames that inform research funding and policy-making. We argue that the task of (post-)ELSI approaches is to take inspiration from the ecological complexity of epigenetics in order to bring more relations, relief and gradient in our ethical and political questions.

## Introduction

Epigenetics focuses on the processes of chemical regulation surrounding (hence the prefix ‘epi’) DNA in organisms. As such, epigenetic studies use a different entry point than DNA structure to understand difference and variation in organisms. Today, epigenetics is an umbrella term referring to a broad set of scientific disciplines. The term is more or less inclusive, depending on the particular focus of research. So, apart from molecular biology and biochemistry, epigenetics may also involve epidemiology, historical research, and sociology or anthropology. Epigenetic studies also use various research designs, ranging from setups like cohort studies and experiments in model organisms to tentative collaborations with social scientists (Niewöhner, 2015; Non and Thayer, 2015). As the conceptions and definitions of ‘epigenetics’ differ even among leading practitioners (Pickersgill et al., 2013, Tolwinski, 2013; Pickersgill, 2016), epigenetics ‘in general’ seems to be communicable and graspable only through broad stroke definitions. Viewed differently, however, the relative imprecision of the term is precisely what holds the area of activity and scientific engagement together, and indeed may explain its success and proliferation over the last decade or so (Meloni and Testa, 2014). Landecker and Panofsky (2013) argue that epigenetics is more accurately described as a shift in *focus* within biomedical sciences, rather than as a new discipline as such. For them, it is about shifting attention from the sequence of genes to the expression of genes, or from “timeless genetic difference to time-dependent gene-regulatory difference” (Landecker and Panofsky, 2013: 340). In this article, we ask to what extent this shift in focus in biomedicine affects the type of questions one can ask about human society.

## Relations Between Normativity and Scientific Evidence

“Paternal obesity is associated with IGF2 hypomethylation in newborns: results from a Newborn Epigenetics Study (NEST) cohort” (Soubry et al., 2013).

The above is the title of a study that was published in *BMC Medicine* in 2013. The research in question was conducted at Duke University. The team collected DNA from umbilical cord blood samples from 79 newborns and investigated methylation at a specific region of the DNA. Multiple regression models were used to determine potential associations between the methylation patterns in specific regions of the DNA, and parental obesity before conception. The authors concluded that:

“While our small sample size is limited, our data indicate a preconceptional impact of paternal obesity on the reprogramming of imprint marks during spermatogenesis. […] our study provides evidence for transgenerational effects of paternal obesity that may influence the offspring’s future health status” (Soubry et al., 2013).

The article was followed by a comment in the same issue with a very straightforward title: “Fat dads must not be blamed for their children’s health problems.” It is particularly interesting to read *why* the ‘fat dads’ must not be blamed:

“It is tempting to over-emphasize the role of a small number of parent-of-origin expressing genes and to speculate about the effects of modest variation in methylation, but we must not be too hasty to blame either parent for their offspring’s health outcomes *without being certain that these effects are consequentially robust*” (Moore and Stanier, 2013, emphasis added).

Does this imply that ‘blaming,’ as the authors call it, is in order as soon as the right amount of evidence from epigenetic research is obtained? Many questions can be asked about what the right amount of evidence is or should be. And of course, a valid operationalization of obesity over an ethnic and socially diverse population is yet another (but related) issue. But we want to ask a different question: why talk about ‘blaming’ in the first place? Let us imagine that the evidence is robust enough, and that researchers agree on the importance of a newly discovered mechanism of epigenetic inheritance. The evidence in question is first and foremost evidence of a *new type of relation* between parents and their offspring. If one thinks of obesity as a condition for which an *individual* is to be held responsible, then one might draw an implication from the newly discovered relation: namely that the responsibility of an obese individual for his or her own condition extends to their offspring. This implication, however, does not follow from epigenetic research itself, but from the conceptions or implicit theories one has about obesity. Framing obesity as an individual responsibility has since long been criticized by sociologists. Empirical studies in sociology show that obesity is prevalent among poor households^1^. For sociologists, this is not simply a matter of poor education and wrong dietary ‘choices,’ but it rather indicates that choices are not the same for everyone. Returning to epigenetics, the example of obesity serves to make the following point: there is no straight logical path from epigenetic evidence, no matter how robust it is considered to be, to the responsibility of an individual. The step to blame or responsibility involves a moralizing framework, based on political and cultural assumptions regarding the individual, the notion of ‘choice,’ and disease etiology (Lupton, 1995).

Recent critical scholarship in the social sciences indicates another problematic issue: not only may scientific evidence nourish pre-existing moral assumptions, as in the example on obesity, but moral assumptions may also nourish the very production of scientific evidence in the research design. Epigenetics, and especially environmental epigenetics, has been receiving increasing attention in social science and science and technology studies (STS) scholarship over the past years. Enthusiasm about the inclusion of the socio-cultural and political factors in biology, exists alongside critical interrogations about how these factors are operationalized in research designs. For example, the inclusion of behavioral and psychological parameters in research designs geared to detect corresponding variations at the biomolecular level, has more than often led to the reification of supposed racial differences, stereotypes, gender-biased conceptions and unwarranted analogies between humans and model organisms. A short review of such issues was recently published by a consortium of researchers in the social and biological sciences in *EMBO Reports* (Müller et al., 2017). In this review, the authors caution against experimental reductionism and the exclusion of social complexity in environmental epigenetics. A similar comment was published in *Nature*, authored by a panel of scientists in the humanities, social and life sciences (Richardson et al., 2014). The comment was entitled “Don’t blame the mothers.” This example puts forward a different reason to refrain from blaming than our example on ‘fat dads’ above. Here, the issue is not a lack of scientific evidence to prove a moral point, but a restricted focus in research on the ‘mother’ as an individual. The authors point to a long history in which women have been held fully responsible for the health of their children. Research designs and science communication tend to reiterate this cultural and gender-biased vision of responsibility and the related policies of social control over women that this vision supports. For example, during the 1980s and 1990s many studies in the US focused on the adverse effects of the pregnant mother’s drug abuse. These studies frame substance abuse as an individual, rather than a social problem and they have provided the basis for repressive action against individuals, leading to prosecution and imprisonment. Current environmental epigenetic research, as many researchers point out, runs the same risks of importing unexamined social and moral bias into science communication and research design itself. Parental responsibility and gender are only a part of a broader set of debates about various other forms of bias and assumptions related to the developmental origins of health and disease (DOHaD), race, class, occupational health, mental health, and criminal behavior (see Meloni et al., 2018 for a rich overview of debates and new concepts).

A key problem is that socially complex phenomena must be made workable – i.e., defined and restricted as measurable parameters and variables – in research designs which remain fundamentally biomolecular in scope. As a consequence, the promising integration of socio-cultural dimensions in biological research seems bound to lead to new forms of reductionism. Researchers argue that this reduction of social realities in molecular terms obliterates them from focus *as* social realities (Warin et al., 2011; Lock, 2013; Richardson et al., 2014; Müller et al., 2017). Interviews with epigenetics researchers in the US point to yet another problem – of institutional nature – making it difficult to seriously address social and political environments: in order to write successful applications for research grants, or to be taken seriously by peers, researchers in environmental epigenetics feel that any ‘environment’ they wish to investigate needs to be designed so that data collection can happen within the human body at the molecular level, even when self-reported data and other types of information may be more indicative of that environment (Darling et al., 2016). This brings another question into play when discussing issues of bias and reductionism in (environmental) epigenetics: where are ‘environments’ and ‘social factors’ defined in the first place? What we deduce from Darling et al. (2016) is that there are cases where research funding opportunities privilege problem-frames that lead to biological reductionism which potentially impoverishes the ways one addresses the social (a well-established point), *but also the ways one addresses the biological*.

## An Epigenetic Prism to Norms and Values

Increasingly, calls are made for collaboration between the life and social sciences in order to equally address the complexities of the social and the biological in epigenetic research. Such calls and the rationale behind them, make a good fit with post-ELSI approaches, even though these approaches may not always be explicitly referred to. Proposals for a post-ELSI program criticize ELSI approaches for various reasons, including “the emphasis it tends to place on a simplified, linear model of innovation, the attention given to the outcomes of research and innovation over practices, the assumption that it is easy to classify outcomes as “negative” or positive,” and the distinction between “science” and “society” that it continues to embed” (Balmer et al., 2015, p.4).

Myskja et al. (2014), however, argue that the post-ELSI approach caricaturizes the ELSI program and disregards the evolutions and progress made in the ELSI field. According to them, the critiques of ELSI have, to a large extent, already been raised and addressed within ELSI itself (and they prefer the EU acronym *ELSA*, with ‘A’ for ‘aspects’) when it evolved from what they call ‘ELSA1’ to ‘ELSA2.’ What we retain from Myskja et al. (2014) is that it is not very helpful to make generalizing claims about ELSI. It is better to appreciate differences between approaches and forms of collaboration, because such differences may have their reasons. The message that we also read in their paper is that the subject-matter at hand should inform discussions about how to best approach it, rather than have funding bodies promote one type of approach, supposedly applicable across the board of technoscience. For example, Rothstein et al. (2009) have provided an extensive overview of possible future ELSI issues in the area of epigenetics. What is appreciable in this overview is the attention these authors give to the specificities of epigenetics, and how these might be expected to entail the re-evaluation of a number of specific legal provisions, legal concepts and existing court rulings. We insist on the legal aspects of their paper because it is here that the specificities proper to epigenetics serve the authors to lay bare a number of concepts and technical complexities that are proper to the realm of law (e.g., issues of liability across generations). Though mostly and necessarily hypothetical, the authors make an attempt at matching the complexities of the two areas: science and law. We now ask the following question – the core question of our paper: can epigenetics prompt us in a similar manner to lay bare some of the assumptions and limits of our moral and normative views? Is this possible, not *despite* but *because of* the fact that moral and normative issues already appear early on in the research process, when a problem is framed and operationalized? A caveat in Rothstein et al.’s (2009) impressive overview is that they seem to make a wager on epigenetic evidence as something that will accumulate and become stronger over time. They reason from there to assess the possible implications. The restricted focus on the implications of research *outcomes* has been an important critique of ELSI programs. For sure, new data and evidence become available all the time, but evidence is not straightforward, as we have seen in the examples above, and it doesn’t always evolve in a cumulative or additive manner (Kuhn, 1962). The point that we want to make, however, is that epigenetics – and any science for that matter – should not be reduced to the data or evidence it provides. Epigenetics, we argue, should also be considered as a way of thinking – a mode of attention toward particular problems in biology and the study of life on Earth. Epigenetics provides perspectives on the environment, inheritance, evolution and even culture, that are different from neo-Darwinian approaches (Jablonka and Lamb, 2005; Landecker and Panofsky, 2013; Jablonka, 2016). Historically, *epigenesis* stands in opposition to models of preformation and it has changed the focus and questions of developmental biology (Haraway, 2004-1976). One characteristic of epigenetics that is particularly salient throughout all the examples we gave is its attention to relationality. Epigenetics constantly tries to draw new relations within individual organisms, and between organisms and their environment. Epigenetics draws relations between areas and entities about which we care as humans. This may happen directly through the study of human cohorts and their life experience, or indirectly through the study of biomolecular mechanisms in model organisms. Epigenetics encourages ecological thinking (Jablonka and Lamb, 2005) and it provocatively interferes with our worldview as it mobilizes morally and culturally coded problems such as illness, exposure and inheritance into its research design. Talk of blame and responsibility should not come as a surprise then. One might argue that research should be purified from all moral bias, but this presupposes that we always already know where, and under what form such bias resides. Rather than trying to purify epigenetics from bias, we propose that epigenetics be used as a prism to diffract and interrogate our own worldviews (must they be human-centered all the time?), moral concepts (what is responsibility? Should we distinguish different forms?) and the basic concepts we use to think society (what is a person? What is an individual? Should there be only one answer to each of these questions?). Recent anthropological field work in Chinese epigenetics labs points to such questions (Lamoreaux, 2016). Lamoreaux discusses that the ‘mother’ is at the center of interest in epigenetic research, but that this doesn’t necessarily nourish any blame-frames as we know them in debates in the West. The mother is not an individual in the Western sense, but a special space of focus and care within a broader ecology and cosmology. Similarly, in the West, we have no trouble seeing plants as part of larger ecosystems. Epigenetic studies on plants show how they adapt to environmental cues, including climate change. What would change in our ELSI debates if we experimented with decentering the human in favor of a broader ecological picture? Interestingly, this question does not imply an exercise in projecting the evolution of epigenetic evidence in order to anticipate its *future* implications, but it requires interrogating our *present* worldviews and concepts. Joly et al. (2016) say that ELSI and other critical scholars tend to focus on hypothetical negative consequences of epigenetics, and they urge for a more nuanced focus on tangible issues in the present, such as the appropriate way of presenting epigenetics in public discussion, without premature risk scenarios and hype. We agree, but the question remains how to present epigenetics ‘appropriately’. Is this only a matter of better science communication? For us, the main challenge is one of *accommodation*. How do we make things fit (*accommodare*) without reductionism, and where does reductionism come from? Critics often refer to biological reductionism, but we find it hard to see how biology reduces anything at all. If anything, biology complexifies things. However, the canalization of research funding to specific types of investigation and problem frames (rather than others), is perhaps a more accurate source of reductionism to pinpoint. ‘Biological’ reductionism touches both the social and the biological. It naturalizes categories of race, gender and class while it reduces the scope of biology to questions that can be readily translated into evidence for policy-making.

## Conclusion

In debates about epigenetics, we propose an additional dimension and a refocusing of attention from issues of scientific evidence alone (asking what kind of evidence epigenetics produces and how it does so) to a broader picture on epigenetics as a mode of attention that encourages relational and process-oriented thinking with entities, values and scales that may not yet fit within conventional problem-frames that inform research funding and policy-making. In an era where research is increasingly expected to be policy-relevant, the provocative relationality of epigenetics may question policies themselves when these are based on categories (individual responsibility, lifestyle choices vs. genetic determinism, the concept of ‘health’ itself) that epigenetics may open up for reconsideration, refinement and enrichment. Such would make epigenetics policy-relevant indeed, without however submitting to the concerns and problem frames of policy makers. This is where (post-)ELSI approaches have a role to play today. Epigenetics currently raises normative and moral questions as we have shown in examples. We argue that the task of (post-)ELSI approaches is to take inspiration from the ecological complexity of epigenetics in order to bring more relations, relief and gradient in our ethical and political questions. In sum, we argue that it is worthwhile trying to think *with* epigenetics and not only ‘about’ it^2^.

## Author Contributions

KH and IVH contributed to this article in content and writing.

## Conflict of Interest Statement

The authors declare that the research was conducted in the absence of any commercial or financial relationships that could be construed as a potential conflict of interest.

## Footnotes

1 http://www.health-inequalities.eu/resources/marmot-reviews/

2 Thinking with Epigenetics was the name and subject of a two-day workshop organized at the University of Leuven in Belgium on 15–16th June 2017.

## References

[B1] Balmer A. S. Calvert J. Marris C. Molynuex-Hodgson S. Frow E. Kearnes M. (2015). Taking roles in interdisciplinary collaborations: reflections on working in post-ELSI spaces in the UK synthetic biology community. *Sci. Technol. Stud.* 28 3–25.

[B2] Darling K. W. Ackerman S. L. Hiatt R. H. Lee S. S.-J. Shim J. K. (2016). Enacting the molecular imperative: how gene-environment interaction research links bodies and environments in the post-genomic age. *Soc. Sci. Med.* 155 51–60. 10.1016/j.socscimed.2016.03.007 26994357 PMC4815914

[B3] Haraway D. (2004-1976). *Crystals, Fabrics, and Fields. Metaphors that Shape Embryos.* Berkeley, CA: North Atlantic Books.

[B4] Jablonka E. (2016). Cultural epigenetics. *Soc. Rev. Monogr.* 64 42–60. 10.1002/2059-7932.12012

[B5] Jablonka E. Lamb M. J. (2005). *Evolution in Four Dimensions. Genetic, Epigenetic, Behavioral and Symbolic Varition in the History of Life.* Cambridge, MA: MIT Press.

[B6] Joly Y. So D. Saulnier K. Dyke S. O. M. (2016). Epigenetics ELSI: darker than you think? *Trends Genet.* 32 591–592. 10.1016/j.tig.2016.07.001 27460649

[B7] Kuhn T. (1962). *The Structure of Scientific Revolutions.* Chicago, IL: Chicago University Press.

[B8] Lamoreaux J. (2016). What if the environment is a person? Lineages of epigenetic science in a toxic China. *Cult. Anthropol.* 31 188–214. 10.14506/ca31.2.03

[B9] Landecker H. Panofsky A. (2013). From social structure to gene regulation, and back: a critical introduction to environmental epigenetics for sociology. *Annu. Rev. Soc.* 39 333–357. 10.1146/annurev-soc-071312-145707

[B10] Lock M. (2013). The epigenome and nature/nurture reunification: a challenge for anthropology. *Med. Anthropol.* 32 291–308. 10.1080/01459740.2012.746973 23768216

[B11] Lupton D. (1995). *The Imperative of Health. Public Health and the Regulated Body.* London: SAGE Publications.

[B12] Meloni M. Cromby J. Fitzgerald D. Lloyd S. (eds). (2018). *The Palgrave Handbook of Biology and Society.* London: Palgrave Macmillan UK 10.1057/978-1-137-52879-7

[B13] Meloni M. Testa G. (2014). Scrutinizing the epigenetics revolution. *Biosocieties* 9 431–456. 10.1057/biosoc.2014.22 25484911 PMC4255066

[B14] Moore E. G. Stanier P. (2013). Fat dads must not be blamed for their children’s health problems. *BMC Med.* 11:30. 10.1186/1741-7015-11-30 23388448 PMC3584737

[B15] Müller R. Hanson C. Hanson M. Penkler M. Samaras G. Chiapperino L. (2017). The biosocial genome? Interdisciplinary perspectives on environmental epigenetics, health and society. *EMBO Rep.* 18 1677–1682. 10.15252/embr.201744953 28931580 PMC5623843

[B16] Myskja B. Nydal R. Myhr A. (2014). We have never been ELSI researchers – There is no need for a post-ELSI shift. *Life Sci. Soc. Policy* 10:9. 10.1186/s40504-014-0009-4 26085445 PMC4648827

[B17] Niewöhner J. (2015). Localizing biology through co-laboration. *New Genet. Soc.* 34 219–242. 10.1080/14636778.2015.1036154 19046038

[B18] Non A. L. Thayer Z. M. (2015). Epigenetics for anthropologists: an introduction to methods. *Am. J. Hum. Biol.* 27 295–303. 10.1002/ajhb.22679 25711975

[B19] Pickersgill M. (2016). Epistemic modesty, ostentatiousness and the uncertainties of epigenetics: on the knowledge machinery of (social) science. *Sociol. Rev. Monogr.* 64 186–202. 10.1002/2059-7932.12020 27397944 PMC4935999

[B20] Pickersgill M. Niewöhner J. Müller R. Martin P. Cunningham-Burley S. (2013). Mapping the new molecular landscape: social dimensions of epigenetics. *New Genet. Soc.* 32 429–447. 10.1080/14636778.2013.861739 24482610 PMC3898699

[B21] Richardson S. S. Daniels C. R. Gillman M. W. Golden J. Kukla R. Kuzawa C. (2014). Society: don’t blame the mothers. *Nature* 512 131–132. 10.1038/512131a 25119222

[B22] Rothstein M. A. Yu C. Marchant G. E. (2009). The ghost in our genes: legal and ethical implications of epigenetics. *Health Matrix* 19 1–62. 19459537 PMC3034450

[B23] Soubry A. Schildkraut J. M. Murtha A. Wang F. Huang Z. Bernal A. (2013). Paternal obesity is associated with IGF2 hypomethylation in newborns: results from a Newborn Epigenetics Study (NEST) cohort. *BMC Med.* 11:29. 10.1186/1741-7015-11-29 23388414 PMC3584733

[B24] Tolwinski K. (2013). A new genetics or an epiphenomenon? Variations in the discourse of epigenetics researchers. *New Genet. Soc.* 32 366–384. 10.1080/14636778.2013.849928

[B25] Warin M. Moore V. M. Zivkovic T. Davies M. J. (2011). Telescoping the origins of obesity to women’s bodies: how gender inequalities are being squeezed out of Barker’s hypothesis. *Ann. Hum. Biol.* 38 453–460. 10.3109/03014460.2011.591829 21696324

